# Elevated C-met in Thymic Dendritic Cells of New Zealand Black Mice

**DOI:** 10.1080/1044667021000003943

**Published:** 2002-03

**Authors:** Tomoyuki Okada, Zhe-Xiong Lian, Tom Hsu, Mitsuru Naiki, Aftab A. Ansari, Dan Robinson, Hsing-Jien Kung, Richard Boyd, M. Eric Gershwin

**Affiliations:** 1 Division of Rheumatology/Allergy and Clinical Immunology University of California at Davis School of Medicine TB 192, One Shields Avenue Davis CA 95616 USA; 2 Institute of Bio-Active Science Nippon Zoki Pharmaceutical Co. Ltd., Kinashi Yashiro-Cho Kato-gun Hyogo 673-1461 Japan; 3 Department of Pathology Emory University School of Medicine Atlanta GA 30322 USA; 4 Basic Science Research UC Davis School of Medicine UC Davis Cancer Center Sacramento California CA 95817 USA; 5 Department of Microbiology and Immunology Monash University Victoria Australia

## Abstract

New Zealand Black (NZB) mice are a well-known animal model of human autoimmune disease. Although the mechanism for development of autoimmunity is unclear, NZB mice are well known for severe thymic microarchitecture abnormalities. It is thought that thymic dendritic cells (DC) may play a role in thymic education and contribute to the autoimmune process. To address this issue and, in particular, that qualitative and/or quantitative differences exist in thymic DC, we took advantage of a novel restriction analysis system that allow definition of differences in the expression of tyrosine kinases using highly enriched populations of thymic DC from NZB compared to BALB/c and C57BL/6 mice. The method chosen, restriction analysis of gene expression, allowed the determination of protein tyrosine kinase transcription profiles. We report herein that NZB mice have a significant upregulation of C-met compared to the control strains. The abnormality of the C-met transcription was confined to thymic DC. We believe that its abnormal expression reflects the resistance of thymic cells to apoptosis, which will ultimately lead to defects and/or abnormal signaling by the interaction of thymic DC and thymocytes. Further studies involving such interactions are under way.


*Developmental Immunology*, 2002 VOL. 9 (1), pp. 29–34

Taylor & Francis healthsciences

### Elevated C-met in Thymic Dendritic Cells of New Zealand Black Mice

TOMOYUKI OKADA[a,b], ZHE-XIONG LIAN[a], TOM HSU[a], MITSURU NAIKI[b], AFTAB A. ANSARI[c], DAN ROBINSON[d], HSING-JIEN KUNG[d], RICHARD BOYD[e] and M. ERIC GERSHWIN[a,*]

[a]*Division of Rheumatology/Allergy and Clinical Immunology, University of California at Davis School of Medicine, TB 192, One Shields Avenue, Davis, CA 95616, USA;* [b]*Institute of Bio-Active Science, Nippon Zoki Pharmaceutical Co. Ltd., Kinashi, Yashiro-Cho, Kato-gun, Hyogo 673-1461, Japan;* [c]*Department of Pathology, Emory University School of Medicine, Atlanta, GA 30322, USA;* [d]*Basic Science Research, UC Davis School of Medicine, UC Davis Cancer Center, Sacramento, California, CA 95817, USA;* [e]*Department of Microbiology and Immunology, Monash University, Victoria, Australia*

New Zealand Black (NZB) mice are a well-known animal model of human autoimmune disease. Although the mechanism for development of autoimmunity is unclear, NZB mice are well known for severe thymic microarchitecture abnormalities. It is thought that thymic dendritic cells (DC) may play a role in thymic education and contribute to the autoimmune process. To address this issue and, in particular, that qualitative and/or quantitative differences exist in thymic DC, we took advantage of a novel restriction analysis system that allow definition of differences in the expression of tyrosine kinases using highly enriched populations of thymic DC from NZB compared to BALB/c and C57BL/6 mice. The method chosen, restriction analysis of gene expression, allowed the determination of protein tyrosine kinase transcription profiles. We report herein that NZB mice have a significant upregulation of C-met compared to the control strains. The abnormality of the C-met transcription was confined to thymic DC. We believe that its abnormal expression reflects the resistance of thymic cells to apoptosis, which will ultimately lead to defects and/or abnormal signaling by the interaction of thymic DC and thymocytes. Further studies involving such interactions are under way.

*Keywords*: C-met; Lupus; Thymic dendritic cells; New Zealand black mice

### INTRODUCTION

The New Zealand Black (NZB) mouse develops an autoimmune disease characterized by high serum IgM levels and autoantibody production that progresses with age (Borchers *et al.*, 2000). Recently our laboratories have shown abnormalities in thymic microarchitecture involving epithelial cell network (Watanabe *et al.*, 1993; Takeoka *et al.*, 1999) and thymic B cells (Taguchi *et al.*, 2001a,b) in the NZB mouse. The mechanisms for these changes and the relationship to autoimmunity are unknown.

Thymic dendritic cells (DC) originate from the bone marrow and reside within the thymic medulla or at the cortico-medullary junction (Ardavin, 1997) and play a pivotal role in thymocyte education, particularly in the context of negative selection (Anderson *et al.*, 1996; Brocker *et al.*, 1997; Ferrero *et al.*, 1997; Brocker, 1999). It has been previously shown that this process of negative/positive selection is, to a large extent, mediated by signals generated intracellularly following lymphoid cell surface interaction with corresponding ligands expressed by cells involved in the selection process, which includes thymic DC. We hypothesized that qualitative and/or quantitative differences may exist in the ability of thymic DC to mediate T cell selection in NZB mice which would contribute to the autoimmune disease in such mice. The recent description of a novel restriction analysis of gene expression (RAGE) (Robinson *et al.*, 1996; Lin *et al.*, 2000), which can be performed on small numbers of cells, prompted us to use this technique for defining differences (if any) in the expression of tyrosine kinases by highly enriched populations of thymic DC from NZB as compared to BALB/c and C57BL/6 mice. The tyrosine kinases were chosen based on an extensive body of data that indicates the importance of such kinases as regulators of intracellular signal-transduction mediating molecules and the mediation of intracellular communication (Blume-Jensen and Hunter, 2001).

We report herein a marked selective thymic DC up-regulation of C-met tyrosine kinase mRNA in NZB mice. This is the first report of an abnormal transcription of a

*Corresponding author. Tel.: +1-530-752-2884. Fax: +1-530-752-4669. E-mail: megershwin@ucdavis.edu

ISSN 1044-6672 print © 2002 Taylor & Francis Ltd
DOI: 10.1080/1044667021000003943

gene that may play an important role in apoptosis and cell–cell adhesion within the NZB thymus. These abnormalities may result in the escape of autoimmune T cells, with development of the resulting disease phenotype.

### MATERIALS AND METHODS

### Mice

BALB/cJ, C57BL/6J, and NZB/BINJ mice were obtained from the Jackson Laboratory (Bar Harbor, ME) and bred at the Animal Resource Services Facility at the University of California, Davis. Four to five weeks-old mice were used for these experiments.

### Antibodies and Reagents

FITC-conjugated rat anti-mouse MHC class II (M5/114.15.2) antibody was purchased from Miltenyi Biotec (Auburn, CA). FITC-conjugated rat anti-mouse CD8α (53-6.7), PE-conjugated hamster anti-mouse CD11c (HL3), FITC and a series of biotin-conjugated rat anti-mouse CD45R/B220 (RA3-6B2), hamster anti-mouse CD80 (16-10A1), rat anti-mouse CD86 (GL1), rat anti-mouse CD40 (3/23) antibodies were each purchased from BD Bioscience (San Diego, CA). Tricolor conjugated streptavidin and anti-mouse CD16/32 antibody were purchased from Caltag laboratories (Burlingame, CA).

### Thymic DC Enrichment

Thymii of mice were subjected to digestion by incubation for 15 min at 37°C in Hank's balanced salt solution (HBSS) containing 60 units/ml collagenase IV (Sigma, St. Louis, MO) and 25 k units/ml deoxyribonuclease I (Sigma) and 0.2% bovine serum albumin (BSA). The resulting population of cells were filtered through a 100 μm mesh, collected by centrifugation at 300*g* for 5 min, and washed with PBS containing 0.2% BSA and 5 mM EDTA. After centrifugation, the cell pellets were resuspended in 17% Optiprep™ (Nycomed Pharma, Oslo, Norway) solution diluted with HBSS (containing 0.2% BSA and 5 mM EDTA) and underlaid in a solution of 12% Optiprep™ in PBS (with 0.2% BSA and 5 mM EDTA) and HBSS (with 0.2% BSA and 5 mM EDTA). The gradient was centrifuged at 600*g* for 15 min at 20°C. The low density cells at the HBSS/12% Optiprep™ solution interface were collected, resuspended in PBS (containing 0.2% BSA and 5 mM EDTA), and recovered by centrifugation.

### Flow Cytometry and Thymic DC Isolation

In efforts to define the frequency and phenotype of the enriched thymic DC, an aliquot of the cell suspension was stained with PE-conjugated anti-mouse CD11c mAb, FITC or biotin-conjugated anti-mouse B220 mAb, and FITC conjugated anti-mouse MHC class II, FITC conjugated anti-mouse CD8α, biotin conjugated anti-mouse CD80 (B7.1), biotin conjugated anti-mouse CD86 (B7.2), or biotin conjugated anti-mouse CD40. Biotinylated antibodies were visualized using tricolor streptavidin. Prior to staining, the FcγII/III receptor on cells were blocked by preincubation of the samples with an anti-mouse CD16/32 antibody to reduce non-specific staining. Cells were incubated with primary antibodies in staining buffer (0.2% BSA and 0.1% $NaN_3$ in PBS) for 30 min at 4°C, washed in washing buffer (0.2% BSA and 5 mM EDTA in PBS), and incubated with tricolor streptavidin in staining buffer for an additional 30 min at 4°C. After the last wash, the cells were resuspended in washing buffer and viable cells from each sample were analyzed using flow cytometry (FACScan, BD) and the data analyzed using Cell Quest software (BD). To isolate thymic DC, thymic $CD11c^{+}B220^{-}$ MHC $classII^{high}$ cells from DC-enriched preparation were collected by 10-parameter MoFlo cell sorter (Cytomation, Fort Collins, CO) equipped with Summit software. The frequency of thymic DC was always $>95\%$ in the preparations utilized for the studies reported herein.

### Splenic DC Isolation

Splenic DC enrichment was performed essentially as described above for thymic DC enrichment. Splenic DC were treated with rat anti-mouse B220 mAb followed by incubation with sheep anti-rat IgG-conjugated magnetic-beads (Dynal, Oslo, Norway). Passage through a magnetic field was used to deplete the $B220^{+}$ cells. Such enriched splenic $B220^{-}$ cells were subsequently incubated with CD11c microbeads (Miltenyi Biotec) and the $CD11c^{+}$ cells isolated and subjected to further enrichment using a cell sorter. The frequency of splenic DC was always $>90\%$ for the assays utilized in the studies reported herein.

### RNA Extraction and cDNAs Synthesis

Total RNA was extracted from splenic and thymic DC from BALB/c, C57BL/6, and NZB mice utilizing the RNeasy kit (QIAGEN, Valencia, CA), cDNAs were synthesized using Superscript™ II (Life Technologies, Grand Island, NY) and oligo $dT_{(12-18)}$ primer (Life Technologies).

### RAGE Analysis for Tyrosine Kinases

The RAGE assay was carried out using a modification of the method previously described (Lee *et al.*, 1999). Essentially, reverse transcription-polymerase chain reaction (RT-PCR) was performed with degenerate primers derived from conserved motifs in the activation loop of the catalytic domains of various tyrosine kinases as follows: primer 1 (sense primer), 5′-AAR RTT DCN GAY TTY GG encoding the amino acid sequence

K[V/I][S/C/G]DFG; primer 2 (antisense primer), 5′-RHA IGM CCA IAC RTC encoding the amino acid sequence DVW[S/A][F/Y]. The mixed bases were defined as follows: N = A + C + T + G, D = A + T + G, H = A + T + C, R = A + G, Y = C + T, M = A + C, and I = deoxyinosine. Primer 1 was labeled with [$\gamma$-$^{33}$P] ATP (NEN Life Science Products, Boston, MA) and T4 polynucleotide kinase (Life Technologies) and PCR was performed by using $^{33}$P labeled primer1, unlabeled primer2, and AmpliTaq Gold DNA polymerase (Perkin–Elmer, Boston, MA). The PCR products were electrophoresed in a 2.4% agarose gel (3:1 ratio of Nusieve GTG and regular agarose; BMA, Rockland, ME). The 153–177 bp bands were excised from the gel and DNA eluted using the QIAEXII gel extraction kit (QIAGEN). Samples were standardized by adjustments based on radioactivity as determined by radioactive scintillation. Equal amounts of radioactive DNA of each sample were digested with AciI, AluI, AvaII, BsrI, DdeI, HaeIII, HinfI, HpaII, MnlI, RsaI, or Taq$^{\alpha}$ I restriction enzyme (New England Biolabs, Beverly, MA) resolved on a 7% DNA sequencing gel, and exposed overnight at −70°C on a fluorescent screen for quantitative analysis on a Phosphoimager® (Molecular Dynamics, Sunnyvale, CA).

### RT-PCR

Serial dilutions of cDNA were PCR-amplified for *c-met*, *Bcl-*$X_L$, or *β-actin* using AmpliTaq Gold DNA polymerase in the presence of either *c-met* primers (5′-CCA GCA GCT TCA GTT ACC GG-3′, 5′-GCG ATG CTG ACA TGC CAC TG-3′) *Bcl-*$X_L$ primer (5′-TGA TTC CCA TGC CAG CAG TGA-3′, 5′-AAC CAC ACC AGC CAC AGT CAT-3′), or *β-actin* primer (5′-CCT AAG GCC AAC CGT GAAAAG-3′, 5′-TCT TCA TGG TGC TAG GAG CCA-3′). Amplified products (*c-met* 182 bp, *Bcl-*$X_L$ 434 bp, *β-Actin* 646 bp) were electrophoresed on a 1.5% agarose gel and visualized with ethidium bromide. In these studies the *c-met* primers were designed using Primer Express 1.0 software (Applied Biosystems, Foster, CA). In addition, the resulting PCR product of 182 bp which corresponds to the expected *c*-met mRNA-derived signals, was confirmed to be the *c*-met gene by direct sequencing of the PCR product.

### Real-Time PCR

Analysis was performed using the GeneAmp 5700 Sequence Detection system (Perkin–Elmer) utilizing SYBR™ Green (Molecular Probes, Eugene, OR) as described (Taguchi *et al.*, 2001a). Primers for *c-met* were the same as used in the RT-PCR experiment. *β-actin* primers (5′-ACT ATT GGC AAC GAG CGG TT-3′, 5′-CAG GAT TCC ATA CCC AAG AAG GA-3′) were designed using Primer Express 1.0 software (Perkin–Elmer). Standard curves were generated using diluted cDNA of thymic CD11c$^+$ cells for *c-met* or *β-actin*. To measure relative intensity between normal and NZB mice, the ratio of *c*-met mRNA level was calculated as *c-met* intensity divided by *β-actin* intensity. All reaction wells were run in duplicate and included control wells without cDNA.

### RESULTS

### Thymic Dendritic Cell Frequency in NZB Mice

Thymii from groups of NZB and for purposes of control, BALB/c and C57BL/6 mice were individually analyzed for the frequency of thymic DC (mean ± SEM). The NZB thymic DC frequency (mean ± SEM) ($4.07 \pm 0.45 \times 10^4/10^8$ total thymocytes) was similar to that of control mice (BALB/c; $3.39 \pm 0.36 \times 10^4/10^8$, C57BL/6; $4.08 \pm 0.35 \times 10^4/10^8$ total thymocytes). The level of expression of cell surface markers in NZB thymic DC was not significantly different from control thymic DC (MHC II$^{high}$, CD8$\alpha^+$, CD40$^+$, B7.1$^+$, B7.2$^+$, data not shown).

### Determination of Protein Tyrosine Kinase Transcription Profile in NZB Thymic DC

We next carried out studies to document the profile of tyrosine kinase utilizing the RAGE assay expressed by highly enriched populations of one month old BALB/c and NZB thymic DC (CD11c$^+$B220$^-$ cells). Amplified PCR products containing a series of tyrosine kinases were digested with the restriction enzymes and the intensity of bands compared. Repeated analysis of visualized bands led to the identification of three bands which had higher signal intensity in NZB thymic DC. Based on the association between the restriction enzyme used and the length of band obtained, the BsrI digested 92 bp band corresponded to the *Frk*, *C-met*, and/or, *Ron* tyrosine kinases (Fig. 1a). The HinfI digest led to a 115 bp band which was identified to be *C-met* tyrosine kinase (Fig. 1b). The RsaI digested 44 bp band corresponded to *Ddr2*, *C-met*, and/or *Trk-B* tyrosine kinases (Fig. 1c). Thus, *C-met* kinase appeared to be the common tyrosine kinase identified by these restriction enzyme digests.

### Identification of PTK mRNA that are Differentially Expressed by NZB Thymic DC

In efforts to confirm the above difference in *c-met* at the mRNA level, RT-PCR analysis was performed using *c-met* specific primers. Figure 2a shows that the *c-met* mRNA level in NZB thymic DC was higher than control strains. To improve sensitivity and quantitation, real-time PCR was performed which included the *β-actin* primers. As seen in Fig. 2b, NZB thymic DC contained a higher level of *c-met* mRNA. Thus, the RT-PCR and real time PCR data concur with the RAGE data and clearly demonstrates that *c-met* mRNA levels in NZB thymic DC is higher than control mice.

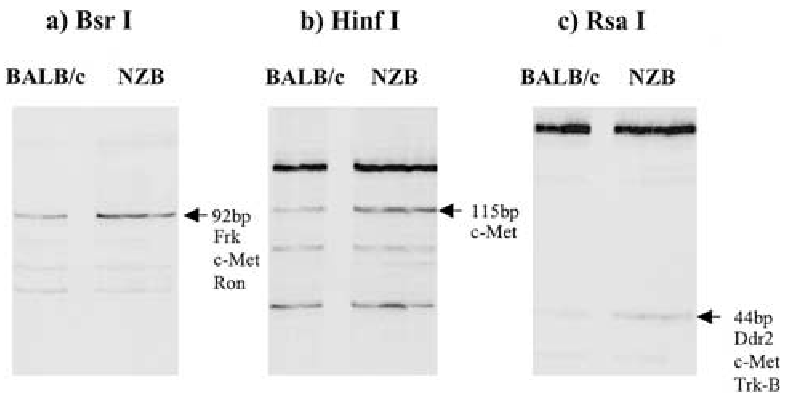

FIGURE 1 Comparison of PTK profile between BALB/c and NZB thymic DC. Sorted thymic CD11$^+$B220$^-$ derived cDNA from BALB/c and NZB mice were amplified using tyrosine kinase primers and digested with BsrI (a), HinfI (b), and RsaI (c). Digested PCR products were electrophoresed and compared with regards to intensity. (a), (b), and (c) showed that *c*-met mRNA may be higher in NZB thymic DC.

### c-met mRNA by Thymocytes and Splenic DC

To ascertain the lineage specificity of the increased expression of *c-met* mRNA, a comparative analysis of *c-met* mRNA levels was carried out on total thymocytes and splenic DC by real time-PCR. Interestingly, thymocytes from control mice and NZB mice had a much lower *c-met* mRNA level (Fig. 3) than corresponding thymic DC. In addition, it was of interest to note that the *c-met* mRNA level in NZB splenic DC was similar to control thymic and splenic DC. These data suggest that the abnormal expression on *c-met* mRNA is restricted to the population of NZB thymic DC and not a global dysregulation of *c-met* expression.

### BCl-$X_L$ mRNA Level in NZB Thymic DC

Previous reports have shown that the interaction of HGF and c-met leads to the induction of resistance to apoptotic cell death (Xiao *et al.*, 2001). To clarify the relationship between c-met upregulation of NZB thymic DC and apoptotic resistance, we examined the mRNA levels of *BCl-$X_L$*, an anti-apoptotic factor, in thymic DC by using RT-PCR. As expected, the *BCl-$X_L$* mRNA level was higher in NZB thymic DC than in control mice (Fig. 4). These data suggest that c-met may function as an integral participant in the anti-apoptotic cascade.

### DISCUSSION

In systemic lupus erythematosus (SLE) patients, the Fyn tyrosine kinase activity of T cells from peripheral blood has been shown to be elevated when compared to healthy donors (Blasini *et al.*, 1998). Using a variety of tyrosine kinase family of mutated or knockout mice, it was reported that such tyrosine kinases are involved in the development of SLE like disease (Morino *et al.*, 1999; Lu

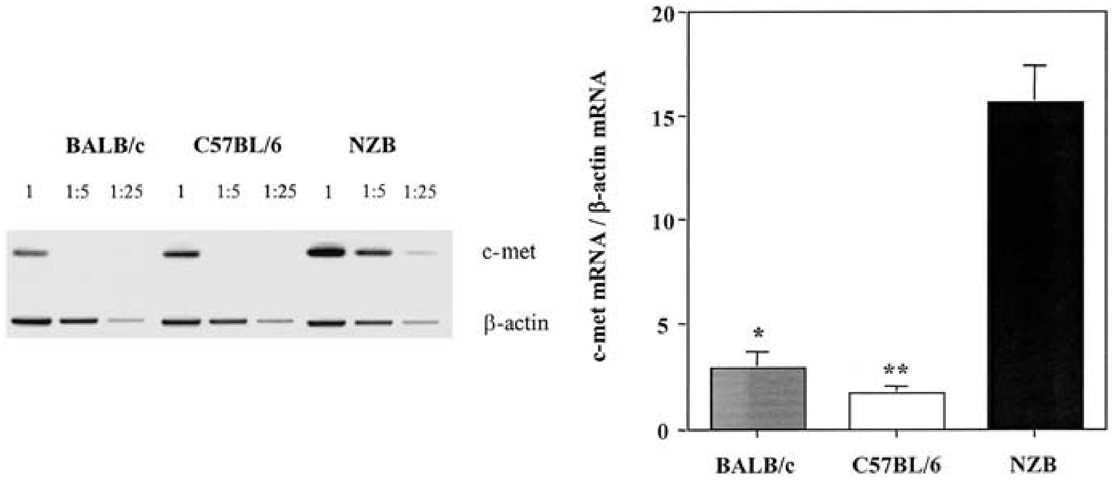

FIGURE 2 Identification of PTK mRNA levels in NZB thymic DC. (a) Serially diluted thymic CD11c$^+$B220$^-$ derived cDNA from BALB/c, C57BL/6, and NZB mouse were amplified using *c-met* or *β-actin* specific primers, electrophoresed, and visualized with ethidium bromide. Intensity of the *c-met* band was higher in NZB thymic DC. (b) Thymic CD11c$^+$B220$^-$ derived cDNA from BALB/c, C57BL/6, and NZB mouse were amplified using *c-met* or *β-actin* specific primers, and visualized with SYBR™ Green by real time-PCR. Standard curves were generated using diluted cDNA of thymic CD11c$^+$ cells for *c-met* or *β-actin*. To measure relative intensity between normal and NZB mice, the ratio of *c-met* mRNA level was calculated as *c-met* intensity divided by *β-actin* intensity.

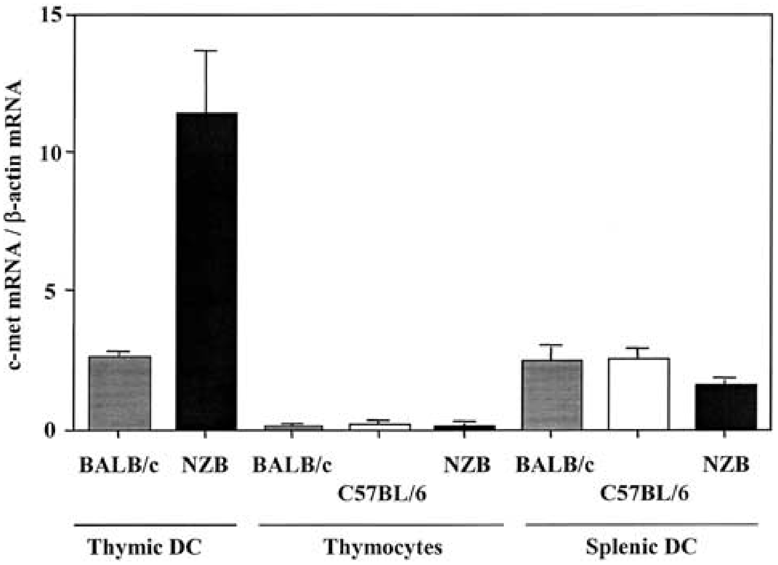

FIGURE 3 C-met mRNA level on thymocytes and splenic DC. Total thymocytes (BALB/c, C57BL/6, and NZB mouse), splenic DC (BALB/c, C57BL/6, and NZB mouse), and thymic DC (BALB/c and NZB mouse) cDNA were amplified by the specific primers *c-met* or *β-actin* using real-time PCR.

and Lemke, 2001; Yu *et al.*, 2001). These data provided the basis for our rationale to screen for all amplified tyrosine kinases in efforts to identify the spectrum of tyrosine kinases that maybe potentially dysregulated in autoimmune prone NZB mice as compared with normal BALB/c and C57BL/6 mice.

C-met kinase is a receptor for the hepatocyte growth factor (HGF) and its gene has been localized to mouse chromosome 6 (Chan *et al.*, 1988). C-met is expressed within a number of cell lineages, including epithelial cells (Di Renzo *et al.*, 1991), endothelial cells (Bussolino *et al.*, 1992), myoblasts (Anastasi *et al.*, 1997), and hematopoietic cells (Galimi *et al.*, 1994; Nishino *et al.*, 1995). Interaction of HGF with c-met activates multiple intracellular signaling pathways involved in muscle and liver formation (Schmidt *et al.*, 1995; Maina *et al.*, 1996), cell proliferation, morphogenesis, and motility. In contrast to thymic DC, *c-met* mRNA levels in NZB splenic DC was similar to control splenic DC, suggesting that either the elevation of *c-met* mRNA levels in NZB thymic DC might be independent of a defect of the entire NZB DC population, or thymic DC may comprise a sublineage of DC with unique homing properties to the thymus.

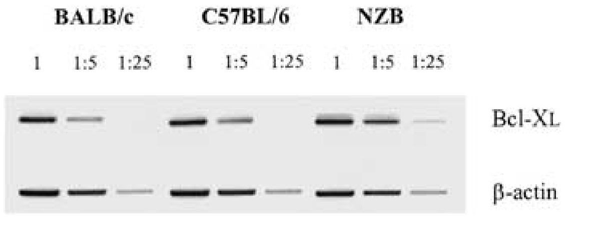

FIGURE 4 *Bcl-$X_L$* mRNA is higher in NZB thymic DC. Serially diluted thymic CD11c$^+$B220$^-$ derived cDNA from BALB/c, C57BL/6, and NZB mice were amplified using *Bcl-$X_L$* or *β-actin* specific primer, electrophoresed, and visualized with ethidium bromide. *β-actin* band intensity replicated the dilution results. The intensity of *Bcl-$X_L$* band was higher in NZB than BALB/c and C57BL/6 thymic DC. Note the intensity of signaling in the NZB sample at 1/5 cDNA dilution, as compared to the lack of expression in control mouse at the same dilution.

It is unknown why the c-met transcription abnormality is confined to the thymic DC population in NZB mice. Among the potential reasons entertained is our previously documented thymic architectural abnormalities in NZB mice (Watanabe *et al.*, 1993; Takeoka *et al.*, 1999). HGF/c-met interaction exerts an upregulation of anti-apoptotic related genes (Aebersold *et al.*, 2001) and downregulation of E-cadherin (Miura *et al.*, 2001) and adhesion molecules, mediated by β-catenin (Behrens *et al.*, 1993; Danilkovitch-Miagkova *et al.*, 2001). From our data, the anti-apoptotic factor, *Bcl-$X_L$* mRNA in NZB thymic DC was significantly higher than control strains of mice. C-met upregulation in thymic DC may contribute to resistance against apoptosis. It is reasoned that the elevation of c-met in NZB thymic DC causes a decrease in the levels of E-cadherin, which leads to defects and/or abnormal signaling by the interaction between thymic DC and thymocytes. It is of interest to note that significant levels of c-met have been documented to be expressed by fetal thymus, liver and hematopoietic cells (Selden *et al.*, 1990; Hu *et al.*, 1993; Galimi *et al.*, 1994; Tamura *et al.*, 1998) but to a lower level by adult thymic and splenic DC in normal mice. Preliminary studies of highly enriched populations of adult thymic CD3$^-$CD4$^-$CD8$^-$ cells (triple negative thymocytes also known as progenitors for T cells and DC) also expressed significantly higher levels of c-met than adult thymocytes (data not shown). These data support the concept that c-met expression may be a marker of immature cells. Thus, such immature NZB thymic DC may not optimally function in the deletion of autoreactive T cells.

It would be important to confirm the relative levels of RNA with levels of c-met protein expression. However, this could not be examined on thymic DC because it was difficult to collect enough cells to carry out such studies. In addition, in efforts to clarify the mechanism of c-met elevation by NZB thymic DC, a study of HGF and adhesion molecules should be performed. Finally, since the data described herein imply the potential of a growth arrest of thymic DC progenitor cells which may be a source of abnormalities in the NZB mouse, they will be closely examined in the context of c-met in future experiments.

### References

Aebersold, D.M., Kollar, A., Beer, K.T., Laissue, J., Greiner, R.H. and Djonov, V. (2001) "Involvement of the hepatocyte growth factor/ scatter factor receptor c-met and of *Bcl-X*$_L$ in the resistance of oropharyngeal cancer to ionizing radiation", *Int. J. Cancer* **96**, 41–54.

Anastasi, S., Giordano, S., Sthandier, O., Gambarotta, G., Maione, R., Comoglio, P. and Amati, P. (1997) "A natural hepatocyte growth factor/scatter factor autocrine loop in myoblast cells and the effect of the constitutive Met kinase activation on myogenic differentiation", *J. Cell Biol.* **137**, 1057–1068.

Anderson, G., Moore, N.C., Owen, J.J. and Jenkinson, E.J. (1996) "Cellular interactions in thymocyte development", *Annu. Rev. Immunol.* **14**, 73–99.

Ardavin, C. (1997) "Thymic dendritic cells", *Immunol. Today* **18**, 350–361.

Behrens, J., Vakaet, L., Friis, R., Winterhager, E., Van Roy, F., Mareel, M.M. and Birchmeier, W. (1993) "Loss of epithelial differentiation and gain of invasiveness correlates with tyrosine phosphorylation of the E-cadherin/beta-catenin complex in cells transformed with a temperature-sensitive v-SRC gene", *J. Cell Biol.* **120**, 757–766.

Blasini, A.M., Brundula, V., Paris, M., Rivas, L., Salazar, S., Stekman, I.L. and Rodriguez, M.A. (1998) "Protein tyrosine kinase activity in T lymphocytes from patients with systemic lupus erythematosus", *J. Autoimmun.* **11**, 387–393.

Blume-Jensen, P. and Hunter, T. (2001) "Oncogenic kinase signalling", *Nature* **411**, 355–365.

Borchers, A., Ansari, A.A., Hsu, T., Kono, D.H. and Gershwin, M.E. (2000) "The pathogenesis of autoimmunity in New Zealand mice", *Semin. Arthritis Rheum.* **29**, 385–399.

Brocker, T. (1999) "The role of dendritic cells in T cell selection and survival", *J. Leukoc. Biol.* **66**, 331–335.

Brocker, T., Riedinger, M. and Karjalainen, K. (1997) "Targeted expression of major histocompatibility complex (MHC) class II molecules demonstrates that dendritic cells can induce negative but not positive selection of thymocytes *in vivo*", *J. Exp. Med.* **185**, 541–550.

Bussolino, F., Di Renzo, M.F., Ziche, M., Bocchietto, E., Olivero, M., Naldini, L., Gaudino, G., Tamagnone, L., Coffer, A. and Comoglio, P.M. (1992) "Hepatocyte growth factor is a potent angiogenic factor which stimulates endothelial cell motility and growth", *J. Cell Biol.* **119**, 629–641.

Chan, A.M., King, H.W., Deakin, E.A., Tempest, P.R., Hilkens, J., Kroezen, V., Edwards, D.R., Wills, A.J., Brookes, P. and Cooper, C.S. (1988) "Characterization of the mouse met proto-oncogene", *Oncogene* **2**, 593–599.

Danilkovitch-Miagkova, A., Miagkov, A., Skeel, A., Nakaigawa, N., Zbar, B. and Leonard, E.J. (2001) "Oncogenic mutants of RON and MET receptor tyrosine kinases cause activation of the beta-catenin pathway", *Mol. Cell Biol.* **21**, 5857–5868.

Di Renzo, M.F., Narsimhan, R.P., Olivero, M., Bretti, S., Giordano, S., Medico, E., Gaglia, P., Zara, P. and Comoglio, P.M. (1991) "Expression of the Met/HGF receptor in normal and neoplastic human tissues", *Oncogene* **6**, 1997–2003.

Ferrero, I., Anjuere, F., MacDonald, H.R. and Ardavin, C. (1997) "*In vitro* negative selection of viral superantigen-reactive thymocytes by thymic dendritic cells", *Blood* **90**, 1943–1951.

Galimi, F., Bagnara, G.P., Bonsi, L., Cottone, E., Follenzi, A., Simeone, A. and Comoglio, P.M. (1994) "Hepatocyte growth factor induces proliferation and differentiation of multipotent and erythroid hemopoietic progenitors", *J. Cell Biol.* **127**, 1743–1754.

Hu, Z., Evarts, R.P., Fujio, K., Marsden, E.R. and Thorgeirsson, S.S. (1993) "Expression of hepatocyte growth factor and *c*-met genes during hepatic differentiation and liver development in the rat", *Am. J. Pathol.* **142**, 1823–1830.

Lee, W.P., Liao, Y., Robinson, D., Kung, H.J., Liu, E.T. and Hung, M.C. (1999) "Axl-gas6 interaction counteracts E1A-mediated cell growth suppression and proapoptotic activity", *Mol. Cell Biol.* **19**, 8075–8082.

Lin, W., Kao, H.W., Robinson, D., Kung, H.J., Wu, C.W. and Chen, H.C. (2000) "Tyrosine kinases and gastric cancer", *Oncogene* **19**, 5680–5689.

Lu, Q. and Lemke, G. (2001) "Homeostatic regulation of the immune system by receptor tyrosine kinases of the Tyro 3 family", *Science* **293**, 306–311.

Maina, F., Casagranda, F., Audero, E., Simeone, A., Comoglio, P.M., Klein, R. and Ponzetto, C. (1996) "Uncoupling of Grb2 from the Met receptor *in vivo* reveals complex roles in muscle development", *Cell* **87**, 531–542.

Miura, H., Nishimura, K., Tsujimura, A., Matsumiya, K., Matsumoto, K., Nakamura, T. and Okuyama, A. (2001) "Effects of hepatocyte growth factor on E-cadherin-mediated cell–cell adhesion in DU145 prostate cancer cells", *Urology* **58**, 1064–1069.

Morino, N., Matsumoto, T., Ueki, K., Mimura, T., Hamasaki, K., Kanda, H., Naruse, T., Yazaki, Y. and Nojima, Y. (1999) "Glomerular overexpression and increased tyrosine phosphorylation of focal adhesion kinase p125FAK in lupus-prone MRL/MP-lpr/lpr mice", *Immunology* **97**, 634–640.

Nishino, T., Hisha, H., Nishino, N., Adachi, M. and Ikehara, S. (1995) "Hepatocyte growth factor as a hematopoietic regulator", *Blood* **85**, 3093–3100.

Robinson, D., He, F., Pretlow, T. and Kung, H.J. (1996) "A tyrosine kinase profile of prostate carcinoma", *Proc. Natl Acad. Sci. USA* **93**, 5958–5962.

Schmidt, C., Bladt, F., Goedecke, S., Brinkmann, V., Zschiesche, W., Sharpe, M., Gherardi, E. and Birchmeier, C. (1995) "Scatter factor/hepatocyte growth factor is essential for liver development", *Nature* **373**, 699–702.

Selden, C., Jones, M., Wade, D. and Hodgson, H. (1990) "Hepatotropin mRNA expression in human foetal liver development and in liver regeneration", *FEBS Lett.* **270**, 81–84.

Taguchi, N., Ansari, A., Hsu, T., Hashimoto, Y., Dorshkind, K., Shultz, L., Naiki, M. and Gershwin, M.E. (2001) "Increased expression of mXBP-1 (TREB-5) in thymic B cells in New Zealand mice", *J. Autoimmun.* **16**, 401–410.

Taguchi, N., Hashimoto, Y., Hsu, T., Ansari, A.A., Shultz, L., Dorshkind, K., Ikehara, S., Naiki, M. and Gershwin, M.E. (2001) "B cells are selectively associated with thymic cortical but not medullary pathology in NZB mice", *J. Autoimmun.* **16**, 393–400.

Takeoka, Y., Taguchi, N., Kotzin, B.L., Bennett, S., Vyse, T.J., Boyd, R.L., Naiki, M., Konishi, J., Ansari, A.A., Shultz, L.D. and Gershwin, M.E. (1999) "Thymic microenvironment and NZB mice: the abnormal thymic microenvironment of New Zealand mice correlates with immunopathology", *Clin. Immunol.* **190**, 388–398.

Tamura, S., Sugawara, T., Tokoro, Y., Taniguchi, H., Fukao, K., Nakauchi, H. and Takahama, Y. (1998) "Expression and function of c-met, a receptor for hepatocyte growth factor, during T-cell development", *Scand. J. Immunol.* **47**, 296–301.

Watanabe, Y., Naiki, M., Wilson, T., Godfrey, D., Chiang, B.L., Boyd, R., Ansari, A. and Gershwin, M.E. (1993) "Thymic microenvironmental abnormalities and thymic selection in NZB.H-2bm12 mice", *J. Immunol.* **150**, 4702–4712.

Xiao, G.H., Jeffers, M., Bellacosa, A., Mitsuuchi, Y., Vande Woude, G.F. and Testa, J.R. (2001) "Anti-apoptotic signaling by hepatocyte growth factor/Met via the phosphatidylinositol 3-kinase/Akt and mitogen-activated protein kinase pathways", *Proc. Natl Acad. Sci. USA* **98**, 247–252.

Yu, C.C., Yen, T.S., Lowell, C.A. and DeFranco, A.L. (2001) "Lupus-like kidney disease in mice deficient in the Src family tyrosine kinases Lyn and Fyn", *Curr. Biol.* **11**, 34–38.

